# Microfat exerts an anti-fibrotic effect on human hypertrophic scar via fetuin-A/ETV4 axis

**DOI:** 10.1186/s12967-023-04065-y

**Published:** 2023-03-31

**Authors:** Qian Yu, Qiang Dai, Zonglin Huang, Chen Li, Li Yan, Xin Fu, Qian Wang, Yi Zhang, Lei Cai, Zhigang Yang, Ran Xiao

**Affiliations:** 1 Research Center, Plastic Surgery Hospital, Chinese Academy of Medical Sciences and Peking Union Medical College, 33 Ba-Da-Chu Road, Beijing, 100144 People’s Republic of China; 2 Key Laboratory of External Tissue and Organ Regeneration, Chinese Academy of Medical Sciences and Peking Union Medical College, Beijing, People’s Republic of China; 3 Department of Burns and Plastic Surgery, Beijing Jishuitan Hospital, Beijing, People’s Republic of China

**Keywords:** Hypertrophic scars, Microfat, Transcription factors, ETV4, Fetuin-A

## Abstract

**Background:**

Hypertrophic scar is a fibrotic disease following wound healing and is characterized by excessive extracellular matrix deposition. Autologous microfat grafting proves an effective strategy for the treatment thereof as it could improve the texture of scars and relieve relevant symptoms. This study aims to explore the potential mechanisms underlying the anti-fibrotic effect of microfat on hypertrophic scars.

**Methods:**

In this study, we injected microfat into transplanted hypertrophic scars in mouse models and investigated the subsequent histological changes and differential expression of mRNAs therein. As for in vitro studies, we co-cultured microfat and hypertrophic scar fibroblasts (HSFs) and analyzed molecular profile changes in HSFs co-cultured with microfat by RNA sequencing. Moreover, to identify the key transcription factors (TFs) which might be responsible for the anti-fibrotic function of microfat, we screened the differentially expressed TFs and transfected HSFs with lentivirus to overexpress or knockdown certain differentially expressed TFs. Furthermore, comparative secretome analyses were conducted to investigate the proteins secreted by co-cultured microfat; changes in gene expression of HSFs were examined after the administration of the potential anti-fibrotic protein. Finally, the relationship between the key TF in HSFs and the microfat-secreted anti-fibrotic adipokine was analyzed.

**Results:**

The anti-fibrotic effect of microfat was confirmed by in vivo transplanted hypertrophic scar models, as the number of α-SMA-positive myofibroblasts was decreased and the expression of fibrosis-related genes downregulated. Co-cultured microfat suppressed the extracellular matrix production of HSFs in in vitro experiment, and the transcription factor ETV4 was primarily differentially expressed in HSFs when compared with normal skin fibroblasts. Overexpression of *ETV4* significantly decreased the expression of fibrosis-related genes in HSFs at both mRNA and protein levels. Fetuin-A secreted by microfat could also downregulate the expression of fibrosis-related genes in HSFs, partially through upregulating *ETV4* expression.

**Conclusions:**

Our results demonstrated that transcription factor ETV4 is essential for the anti-fibrotic effect of microfat on hypertrophic scars, and that fetuin-A secreted by microfat could suppress the fibrotic characteristic of HSFs through upregulating *ETV4* expression. Microfat wields an alleviative influence over hypertrophic scars via fetuin-A/ETV4 axis.

**Supplementary Information:**

The online version contains supplementary material available at 10.1186/s12967-023-04065-y.

## Background

Hypertrophic scar is a fibrotic disorder following wound healing, where hypertrophic scar fibroblasts (HSFs) are stimulated to express excessive fibrosis-associated genes, including alpha-smooth muscle actin (α-SMA), type I and III collagens (COL1 and COL3); however, the production of matrix metalloprotease 1 (MMP-1) that serves as an effective anti-fibrogenic agent is downregulated [1, 2]. Strategies like compression garments, topical products, steroids, and laser therapy have been employed clinically, alone or combined, to treat hypertrophic scars [3], yet they yield unsatisfactory effect of eliminating excessive scar tissue or remolding scars to normal tissue. In recent years, autologous microfat grafting has been applied in the treatment of hypertrophic scars; microfat can not only improve the texture of scar tissue, but also relieve intolerable itching, pain, and other symptoms [4, 5]. Bruno et al. [6] compared the histologic changes of scar tissue at 3 and 6 months postoperatively of adipose tissue injection, and found that the arrangement of collagen became more organized with parallel fibers and the dermal papillae were better vascularized. Consistent with the clinical investigation, the anti-scarring effect of adipose tissue and adipose-derived stromal vascular fraction (SVF) was confirmed on animal models. Wang et al. [7] injected SVF into hypertrophic scars in rabbit ears and found that SVF could reduce the fibrotic tendency in hypertrophic scars, which was manifested as a decreased level of α-SMA-positive myofibroblasts and collagen deposition. However, the molecular mechanisms underlying the anti-fibrotic effect of microfat on hypertrophic scars remain to be elucidated.

Adipose tissue is an essential metabolic and endocrine organ that can secrete a variety of proteins including hormones, growth factor, cytokines, chemokines, etc. The first adipokine, leptin, was reported in 1994 [8]; ever since, hundreds of adipokines have been found in human adipose tissue. Adipokines exert crucial roles in various physiological and pathological processes through autocrine, paracrine and endocrine signaling [9, 10]. With the development of proteomics technology, adipose secretome has already become one of the research hotspots in the field of adipose biology, obesity and its comorbidities. The most common adipokines include adiponectin, resistin, leptin, visfatin, chemerin, vaspin, cytokines (IL-6, TNF-α), coagulation factors (PAI-1), growth factors (VEGF, TGF-β) and complement system proteins (adipsin) [11]. Recent study [12] summarized that adiponectin has strong-antifibrotic properties while leptin acts as a profibrogenic molecule. Adiponectin, an important protein exclusively secreted by adipose tissue, was reported to regulate cutaneous wound healing and act as a vital regulator of the progression of inflammation. Xu et al. [13] reported that decreased expression of adiponectin might be partially responsible for alcohol-induced liver injury in mice, and that adiponectin treatment significantly attenuated inflammation of both alcoholic and nonalcoholic fatty liver diseases in mice as was indicated by the suppression of TNF-alpha level. Leptin is reported to be indispensable for the development of fibrosis, and plays a pivotal role in the activation of hepatic stellate cells which have been identified as major collagen-producing cells in the injured liver [14]. Therefore, it is imperative to further investigate more proteins secreted by adipose tissue to comprehensively understand the anti-scar effect thereof.

Transcription factors (TFs) play an essential role in adult tissue homeostasis and are dysregulated in many diseases [15]. Transcription factors are indispensable in the regulation of genetic activities ensuring that specific genes be expressed in the desired cells at the right time and in the right amount [16]. It has been demonstrated that TF induction can differentiate stem cells, reprogram somatic cells into pluripotency, and transdifferentiate cells between lineages [17]. Doeser MC et al. [18] reported that transient induction of transcription factors OCT4, SOX2, KLF4, and C-MYC in incisional wounds diminished fibrotic activity and led to a reduction in scar tissue formation during wound healing with downregulated profibrotic marker genes like transforming growth factor beta 1 (*Tgf-β1*), *Collagen I*, and vascular endothelial growth factor (*Vegf*). The transcription of collagen genes is regulated by several TFs, whose abnormal expression is the major molecular change found in hypertrophic scars. Previous studies showed that TFs including SP1, AP1, and SMAD play a promoting role in the transcription of *Collagen I*, while NF-κB exerts an inhibitory effect [19–22]. Therefore, it is of great importance to investigate how TFs regulate the collagen synthesis in HSFs.

In our study, we observed the changes of the hypertrophic scars after fat grafting by constructing a hypertrophic scar model in nude mice. At the same time, the co-culture experiment of microfat and HSFs was conducted in vitro, and the effect of microfat on gene expression levels, especially on that of the TFs of HSFs, was explored by transcriptome sequencing. Finally, the protein profiles of microfat and HSFs were detected to identify the vital anti-fibrotic protein secreted by microfat. Our results identified the key TF in HSFs, the microfat-secreted anti-fibrotic adipokine, and the relationship thereof, which not only elucidate the molecular mechanisms underlying the application of microfat in the treatment of hypertrophic scars, but also provide new targets for future drug development.

## Methods

### Microfat harvesting

Microfat tissues were collected from patients via standard vacuum-assisted liposuction in Plastic Surgery Hospital, Chinese Academy of Medical Sciences, and Peking Union Medical College (Beijing, China). The abdomen was the primary donor site, although other areas such as flanks or thighs could also be selected depending on the patient’s preferences. Under local anesthesia, the donor area was incised with a 15-blade scalpel and infiltrated with a modified Klein solution containing 800 mg/L lidocaine and 1:1,000,000 unit dilution of adrenaline. The microfat was harvested with a 3-mm multiport cannula with several side holes of a 1-mm diameter connected to a 20-mL syringe. The plunger of the syringe was pulled back only a few milliliters to create low negative pressure because excessive pressure could rupture cell components in adipose tissue. Upon completion of fat harvesting, the incisions were closed with interrupted sutures. The microfat was rinsed with sterile phosphate-buffered saline solution (PBS) in closed syringes. During approximately 10 min of decantation, the microfat naturally separated into 3 layers. The superior oily and inferior fluid layers were discarded; the middle layer was collected for further processing.

### Isolation and culture of fibroblasts

Hypertrophic scars and adjacent full thickness normal skin tissues were obtained from patients who underwent plastic surgery without previous treatment for scarring in Plastic Surgery Hospital, Chinese Academy of Medical Sciences, and Peking Union Medical College (Beijing, China). Briefly, the tissues were minced and then immersed in 0.25% dispase II (Roche Diagnostics, Indianapolis, IN, USA) dissolved in Dulbecco’s modified Eagle medium (DMEM; Gibco, Gaithersburg, MD, USA). Hypertrophic scars and normal skin tissues were incubated overnight at 4 °C to enzymatically separate epithelial tissue. The next day, the dermis was mechanically isolated from the surrounding scar tissue with forceps and subjected to another round of enzymatic digestion with 0.2% collagenase I (Sigma-Aldrich, St Louis, MO, USA) in DMEM for 2–3 h at 37 °C on a rotator. The digested cell suspension was filtered through a 70-µm cell strainer (Falcon, BD Biosciences, SanDiego, CA) followed by centrifugation at 300*g* for 5 min. Pelleted fibroblasts were resuspended in DMEM containing 10% fetal bovine serum (FBS, GIBCO), 100 units/mL penicillin, and 100 µg/mL streptomycin and cultured at 37 °C with 5% CO_2_ until they reached 80% confluence. Confluent cells were then subcultured at a ratio of 1: 3. Cells of 2–3 passages were used in the following experiments.

Usage of human adipose tissues and fibroblasts in this study was approved by the Institutional Review Board of Plastic Surgery Hospital, Chinese Academy of Medical Sciences, and Peking Union Medical College, and the written consent forms were collected from all tissue donors who agreed to participate in the study.

### Indirect co-culture of HSFs and microfat

HSFs were co-cultured with microfat using the 6-well Millicell® cell culture system (Millipore, Billerica, MA, USA) as previously described. In brief, 1 × 10^5^ HSFs were seeded into the lower chamber of a 6-well with DMEM containing 10% FBS. After the cells reached approximately 80%, the culture media was replaced with a serum-free RPMI 1640 medium. Then, 1ml of microfat were seeded into the upper chamber of the transwell (Millipore, Billerica, MA, USA), while HSFs or microfat were mono-cultured as control. After culture for 2 days, two groups of fibroblasts and their supernatants were harvested for further investigations.

### Cell proliferation assay

The proliferation of HSFs co-cultured with or without microfat was assessed using the cell counting kit-8 (CCK-8 kit; Dojindo, Kumamoto, Japan) according to the manufacturer’s instructions. Briefly, the cells were seeded in 24-well plates at a density of 8 × 10^4^ cells/well. Six replicates were made for each concentration, and the medium was changed every 2 days. After treatment for 1, 2, 3, 4, 5, and 6 days, 100 µL CCK-8 solution was added into each well and the plate was incubated for 2 h at 37 °C. Absorbance was then quantified using a microplate reader (Molecular Devices, Sunnyvale, CA, USA) at 450 nm.

### Scratch wound healing assay

A scratch wound assay was used to evaluate cell migration as previously described. First of all, fibroblasts were seeded in 6-well plates with and without co-culture of microfat. When reached confluence, cells were dealt with serum depleted medium for another 12 h. A scratch wound was then made in the middle of each well by 200-µL pipette tips. After washing three times with PBS, the gap of the scratch was recorded at 0 and 24 h by an inverted phase microscope (Nikon, Japan). Finally, using the Image-Pro Plus system to analyze the wound area. Three independent fields were analyzed in each well, and each experiment was performed in triplicate.

### Knockdown and overexpression experiments

For overexpression, the open reading frame (ORF) of *ETV4*/*C-MYC* was inserted into PCDH-CMV-MCS-EF1-copGFP (System Biosciences, Mountain View, CA), which can encode full-length *ETV4* (PCDH-ETV4-GFP) or *C-MYC* (PCDH-C-MYC-GFP) individually. For knockdown, the HSFs were transfected with small interfering RNAs (siRNAs) targeting *ARID5B*, *SOX4*, *TSC22D3*, and *MEF2C*, respectively, and the hairpin oligonucleotides specific to human *ETV4* was cloned into the PLKO.1 vector. The ORF sequences of ETV4 and C-MYC, siRNAs sequences, and short hairpin RNA (shRNA) sequence targeting *ETV4* were as listed in Additional file 1: Table S1.

For viral packaging, the vectors were transfected into 293T cells together with psPAX2 and pMD2.G (Addgene, Cambridge, MA) using jetPRIME® Transfection Reagent (PolyPlus-Transfection®, Strasbourg, France). The supernatants containing viruses were collected, concentrated, and titered before infecting HSFs with polybrene (5 ug/ml; Millipore, Burlington, MA). At 72 h after transfection, the cells were harvested for further analysis.

### Fetuin-A treatment

The HSFs were seeded into 6 well plates at a density of 2 × 10^5^ cells/well. After 24 h, the cells were treated with 100ug/ml fetuin-A (10,318-H08H, Sino Biological Inc., Minneapolis, MN, USA) for additional 2 days before collection for analysis.

### Real-time quantitative PCR

Total RNA was extracted from cells using TRIZOL reagent (Invitrogen, Grand Island, NY), and 1000 ng of RNA was reverse transcribed using M-MLV reverse transcriptase (Promega, Madison, WI). Real-time PCR was performed using the Fast SYBR Green Master Kit and Light Cycler 96 system (Roche, Basel, Switzerland), and the expression level of each gene was normalized to the expression of the housekeeping gene GAPDH. The primer sequences are listed in Additional file 2 : Table S2.

### Western blot analysis

Total protein was extracted from cells in RIPA buffer on ice. Protein concentrations were determined using a BCA Protein Assay kit (Beyotime, Beijing, China). Equivalent amounts of protein were separated by 10% SDS-PAGE and transferred to a polyvinylidene difluoride membrane (Millipore, Bedford, MA, USA). The membrane was then incubated at 4 °C overnight with primary antibodies against collagen I (COL1, ab34710, Abcam, Cambridge, U.K.), alpha-smooth muscle actin (α-SMA, ab7817, Abcam, Cambridge, U.K.), matrix metalloprotease-1 (MMP-1, ab134184, Abcam, Cambridge, U.K.), and GAPDH (TA-08, ZSGB-BIO, Beijing, China.). Next, the membrane was incubated with the appropriate horseradish peroxidase-conjugated secondary antibodies at room temperature for 2 h and SuperSignal® West Pico Trial Kit (Thermo Fisher Scientific) was applied for protein detection.

### RNA-sequencing and data analyses

RNA integrity was assessed using the RNA Nano 6000 Assay Kit of the Bioanalyzer 2100 system (Agilent Technologies, CA, USA). Sequencing libraries were generated and sequenced by Novogene Corporation (Beijing, China). The sequencing quality of the raw data of the FASTQ format was assessed with FastQC. Low-quality data were subsequently filtered using NGSQC, and all the downstream analyses were based on the data with high quality. Transcriptomes were assembled, and fragments per kilobase per million reads (FPKM) for each gene were computed with StringTie software. The differential expressed genes (DEGs) were analyzed using the DESeq2 R package (1.20.0) and edgeR (3.22.5), and the genes were defined as differentially expressed when |log2FC|≥1 and the p ≤ 0.05. Functional annotation and enrichment analyses were performed using clusterProfiler R package.

### Gene set enrichment analysis

All the expressed genes were pre-ranked by Signal2Noise (the difference of means between CO and CTRL scaled by the SD). Then, the ranked gene list was imported into the GSEA software (version: 4.0.1) (Subramanian et al., 2005). A false discovery rate q < 0.05 was considered to be statistically significant. Precompiled gene sets including REACTOME pathways and GENE ONTOLOGY biological processes in MSigDB (version: 7.0) (Liberzon et al., 2015) were used in this analysis. The results were visualized using the EnrichmentMap plugin of Cytoscape.

### Mass spectrometry and data analyses

Desalted peptide mixture of the supernatants was loaded onto an Acclaim PePmap C18-reversed phase column (75 μm×2 cm, 3 μm, thermo scientific) and separated with a reversed-phase C18 column (75 μm×10 cm, 5 μm, Agela Technologies) mounted on a Dionex ultimate 3000 nano LC system. Peptides were eluted using a gradient of 5–80% (v/v) acetonitrile in 0.1% formic acid over 45 min at a flow rate of 300 nL/min combined with a Q Exactive mass spectrometer (Thermo Fisher Scientific, MA, USA). The eluates were directly entered Q-Exactive MS (Thermo Fisher Scientific, Waltham, MA, USA), setting in positive ion mode and data-dependent manner with full MS scan from 350 to 2000 m/z, full scan resolution at 70,000. Only proteins with scores corresponding to p < 0.05 and with at least two independent peptides were considered. Visualization of protein interaction networks was performed with the String database using a high confidence interaction score (0.7) and experiments, databases, gene fusion, and neighborhood as active interaction sources. GO enrichment analysis was performed using the open access platform Term Finder. Venn diagrams were generated using online tools (http://bioinformatics.psb.ugent.be/webtools/Venn/).

### Statistical analysis

Statistical testing was performed using GraphPad Prism software (GraphPad Software, La Jolla, CA). The data from at least three independent experiments are presented as the means ± standard error of the mean (SEM). Significance was determined with a one-sample t-test followed by Bonferroni correction for multiple testing; *p ≤ 0.05 and **p ≤ 0.01. Data are presented as the means ± SEM. Statistical analysis was performed by sample-independent Student’s t-test and Paired t-test using SPSS16.0 software; a value of p < 0.05 was considered statistically significant.

## Results

### Microfat exerts an anti-fibrotic effect on human hypertrophic scars

**Fig. 1:**
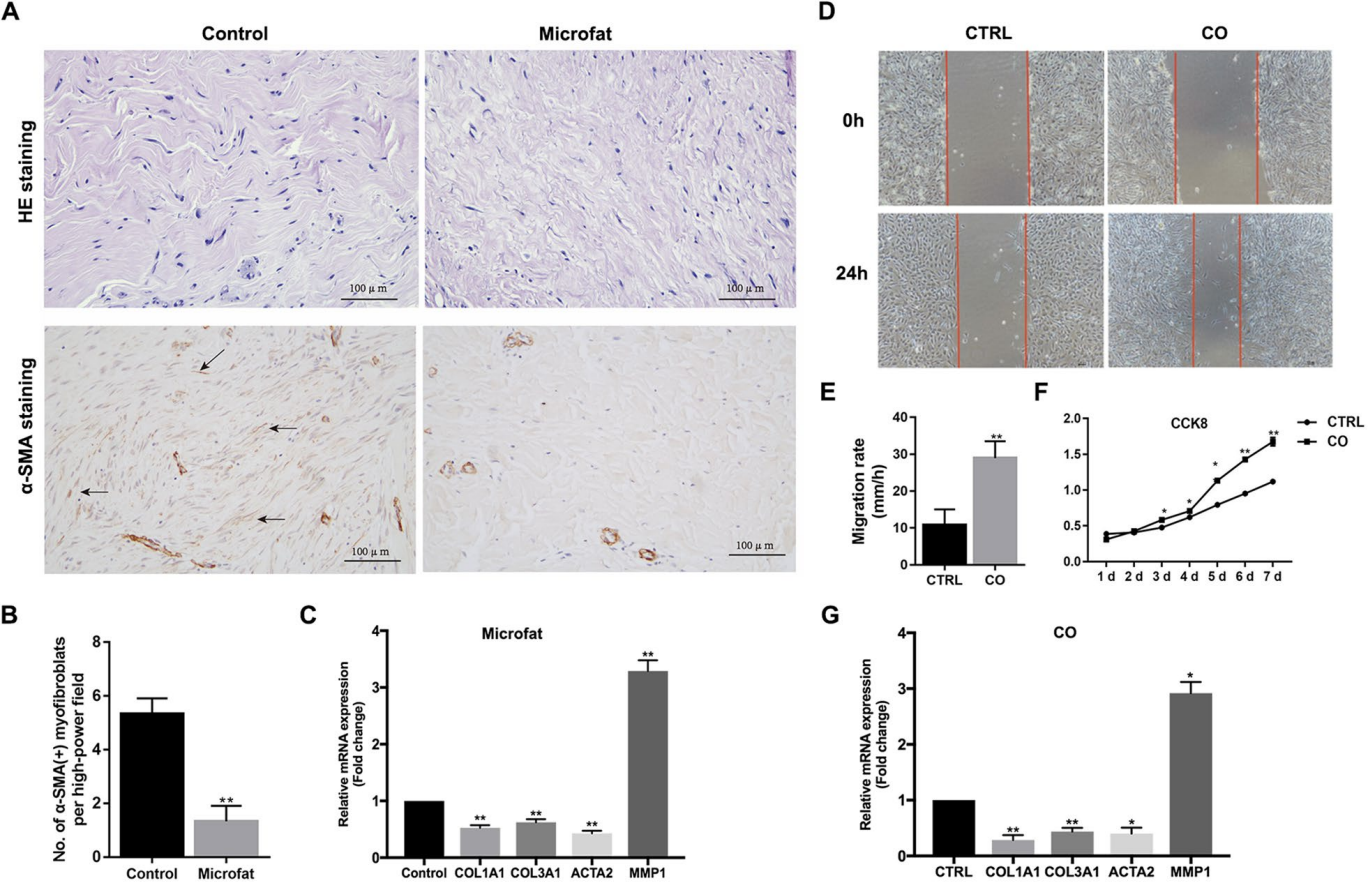
The anti-fibrotic effect evaluation of microfat on human hypertrophic scars both in vivo and in vitro. **A** Histological staining of human hypertrophic scars which were transplanted to nude mice at four weeks after microfat injection. Black arrows indicate α-SMA-positive staining cells. **B** Number of α-SMA-positive myofibroblasts per high-power field. **C** The mRNA expression levels of fibrosis-related genes of human hypertrophic scars after microfat injection were detected by qRT-PCR. **D** Scratch images of HSFs alone (CTRL) and which co-cultured with microfat (CO) after 0 and 24 h. Red arrows draw the margin of scratch lines. **E** Migration rate of the HSFs in CTRL and CO groups. **F** CCK8 assay results of the HSFs in CTRL and CO groups. **G** The mRNA expression levels of fibrosis-related genes of HSF after co-cultured with microfat were detected by qRT-PCR. scale bar = 100 μm. Data are presented as the mean ± SD. *P < 0.05; **P < 0.01

The anti-fibrotic effect of fat grafting on hypertrophic scars has been reported to be clinically effective. To investigate the underlying mechanisms, we injected microfat into human hypertrophic scars which had been subcutaneously transplanted to nude mice, and harvested scar tissues after four weeks. Histological staining showed looser and better-organized collagen fibers in the microfat-treated hypertrophic scars than that in the control group (Fig. 1A). Additionally, the number of α-SMA-positive myofibroblasts was significantly decreased in the microfat-treated group (Fig. 1B). Consistently, the mRNA expression levels of fibrosis-related genes, including *ACTA2*, *COL1A1*, and *COL3A1*, were significantly lower in the microfat-treated group (Fig. 1C), whereas *MMP1* showed a remarkably higher expression. These results confirmed the anti-fibrotic effect of microfat on hypertrophic scars in vivo.

To further explore the influences microfat exerts on the characteristics of HSFs, CCK8 assay and scratch healing experiments were performed after segregated co-culture of HSFs and microfat in vitro. Compared to control cells, co-cultured HSFs exhibited a faster fibroblast migration capability and a higher cell proliferation ability (Fig. 1D, F). Moreover, fibrosis-related genes *COL1A1*, *COL3A1*, and *ACTA2* were significantly downregulated while *MMP1* was significantly elevated in co-cultured HSFs (Fig. 1G), indicating that microfat might perform the anti-fibrotic function by secreting bioactive adipokines.

### Extracellular matrix production turns moderate in HSFs co-cultured with microfat

**Fig. 2:**
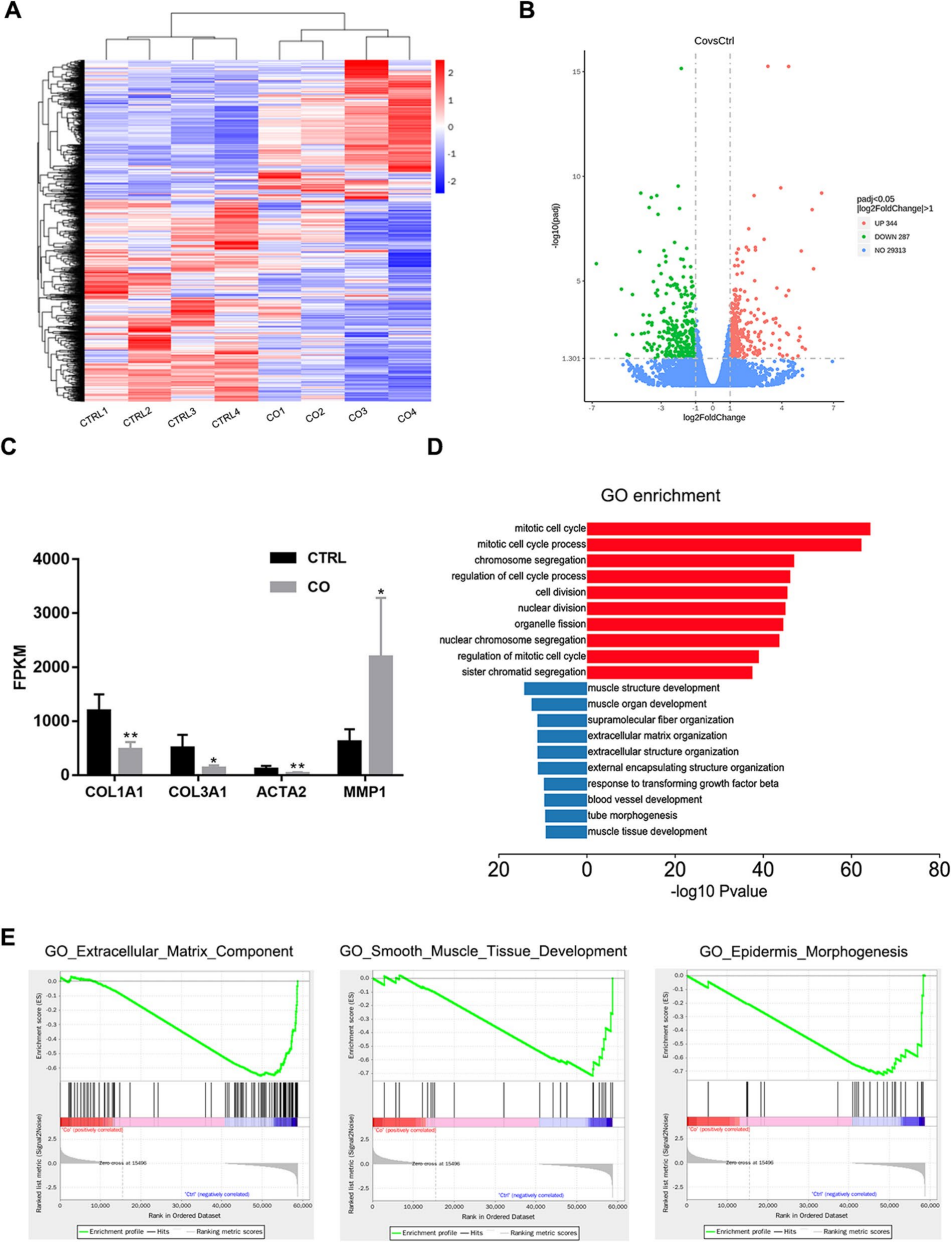
Transcriptome analysis of HSFs co-cultured with microfat. **A** Heatmap of gene expression data between HSFs in CTRL and CO groups. **B** Volcano plot of differential expressed genes. **C** FPKM values of representative fibrosis-related genes expression in CTRL and CO. **D** GO enrichment analysis of up-regulated and down-regulated genes in HSFs between CTRL and CO. **E** GSEA enrichment plots for representative signaling pathways downregulated in CO compared with CTRL. Data are presented as the mean ± SD. *P < 0.05; **P < 0.01

We also analyzed molecular profile changes in HSFs co-cultured with microfat by RNA sequencing. Hierarchical clustering distinguished a significant differential gene expression profile based on the comparison between co-cultured HSFs (CO) and HSFs alone (CTRL) (Fig. 2A). The mean value of the normalized read count values in each group was used to calculate the mRNA expression ratio between CTRL and CO cells. We identified 631 DEGs, of which 344 were upregulated and 287 downregulated in the CO group (Fig. 2B). Additionally, the expression levels of *COL1A1*, *COL3A1*, *ACTA2*, and *MMP1* showed similar changes with the above real-time PCR results of co-cultured HSFs (Fig. 2C).

To provide a cohesive view of the biological functions associated with the changes in gene expression profile in the CO group, we conducted a gene ontology analysis using the Metascape database and the top 10 significantly enriched terms sorted according to *p*-value were shown (Fig. 2D). The upregulated genes showed a strong association with the mitotic cell cycle as well as cell division, which was consistent with the increased proliferation of co-cultured HSFs. The categories enriched among the downregulated genes were mainly associated with muscle development, extracellular matrix organization, and response to TGF-β which is the primary driving factor of fibrosis in most cases. Gene Set Enrichment Analysis (GSEA) also presented that the extracellular matrix component, smooth muscle tissue development, and epidermis morphogenesis were downregulated in the CO group (Fig. 2E). Collectively, these results indicate that the co-culture with microfat could notably reprogram HSFs into a healthier phenotype with appropriate production of extracellular matrix.

### Microfat rescues the dysregulated expression of TFs in HSFs

**Fig. 3:**
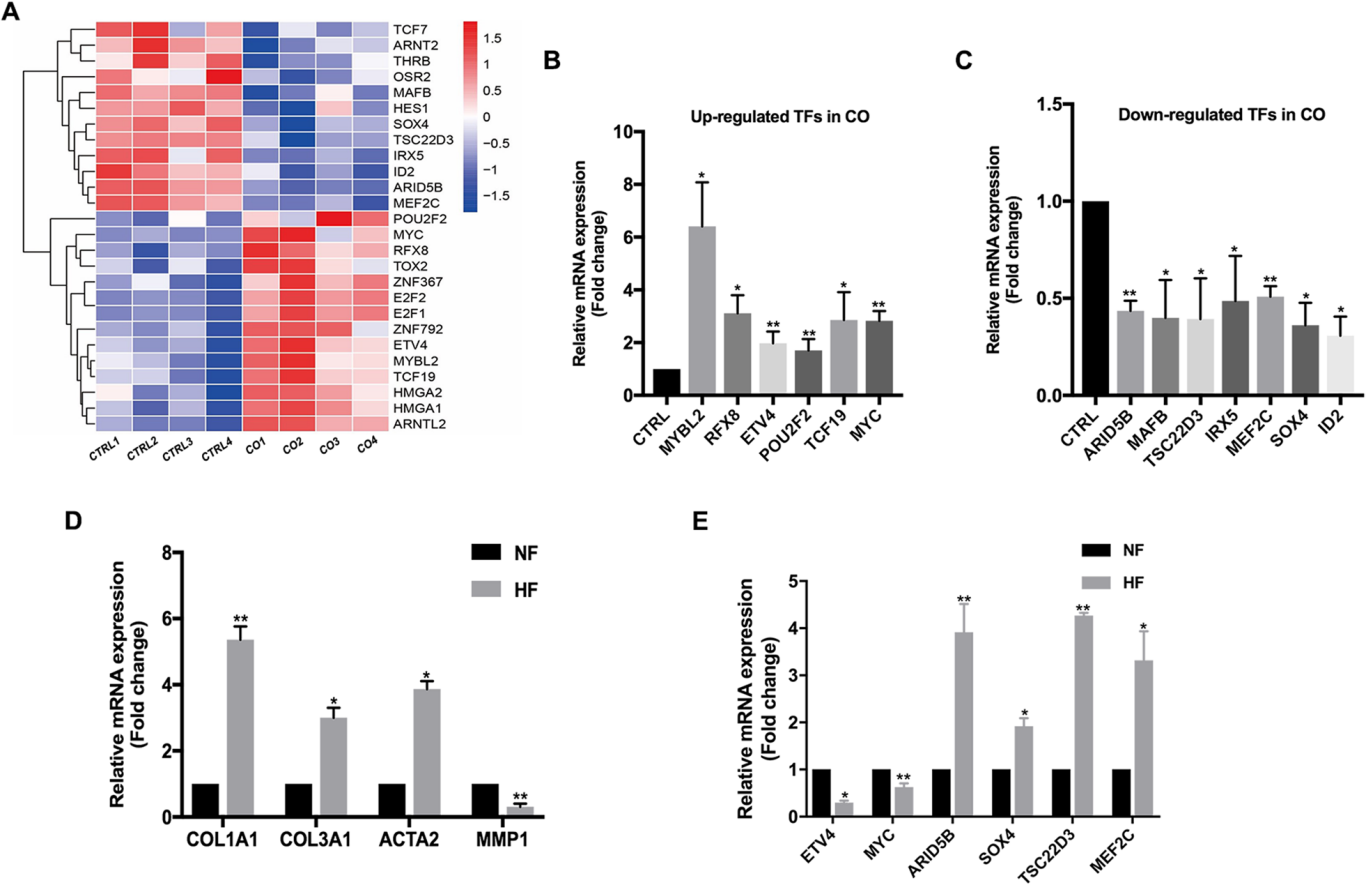
Screening of transcription factors in HSFs involved in the anti-fibrotic effect of microfat. **A** Heatmap of differential expressed TFs in CTRL and CO groups. **B** Upregulated TFs in CO group were validated by qRT-PCR. **C** Downregulated TFs in CO group were validated by qRT-PCR. **D** The mRNA expression levels of representative fibrosis-related genes in scar fibroblasts (SF) and normal skin fibroblasts (NF). **E** The dysregulated TFs in CO group were validated between NF and SF. Data are presented as the mean ± SD. *P < 0.05; **P < 0.01

Several TFs have been reported to play critical roles in regulating fibrogenic activity, therefore, we aim to identify significant TFs in reprogramming HSFs. We found 26 TFs differentially expressed in HSFs co-cultured with microfat, of which 14 were upregulated and 12 downregulated (Fig. 3A). Thirteen TFs, including upregulated *MYBL2*, *RFX8*, *ETV4*, *POU2F2*, *TSF19*, *C-MYC*, and downregulated *ARID5B*, *MAFB*, *TSC22D3*, *IRX5*, *MEF2C*, *SOX4*, *ID2*, were further validated by qRT-PCR (Fig. 3B, C).

To further distinguish the key TF responsible for the anti-fibrogenic effect of microfat, we investigated whether these TFs were primarily changed in HSFs by comparing HSFs with normal skin fibroblasts. HSFs showed increased expression of fibrogenic genes *COL1A1*, *COL3A1*, *ACTA2* and decreased *MMP1* (Fig. 3D); the expression of *ARID5B*, *SOX4*, *TSC22D3*, and *MEF2C* was higher in HSFs than in normal skin fibroblasts, while the expression of *ETV4* and *c-MYC* was lower in HSFs than in normal skin fibroblasts (Fig. 3E). These findings suggested that the dysregulated expression of *ARID5B*, *SOX4*, *TSC22D3*, *MEF2C*, *ETV4* and *C-MYC* in HSFs was rescued by microfat, which probably played crucial roles in the anti-fibrotic function of microfat.

### Upregulated *ETV4* in co-cultured HSFs is responsible for the anti-fibrotic effect of microfat

**Fig. 4:**
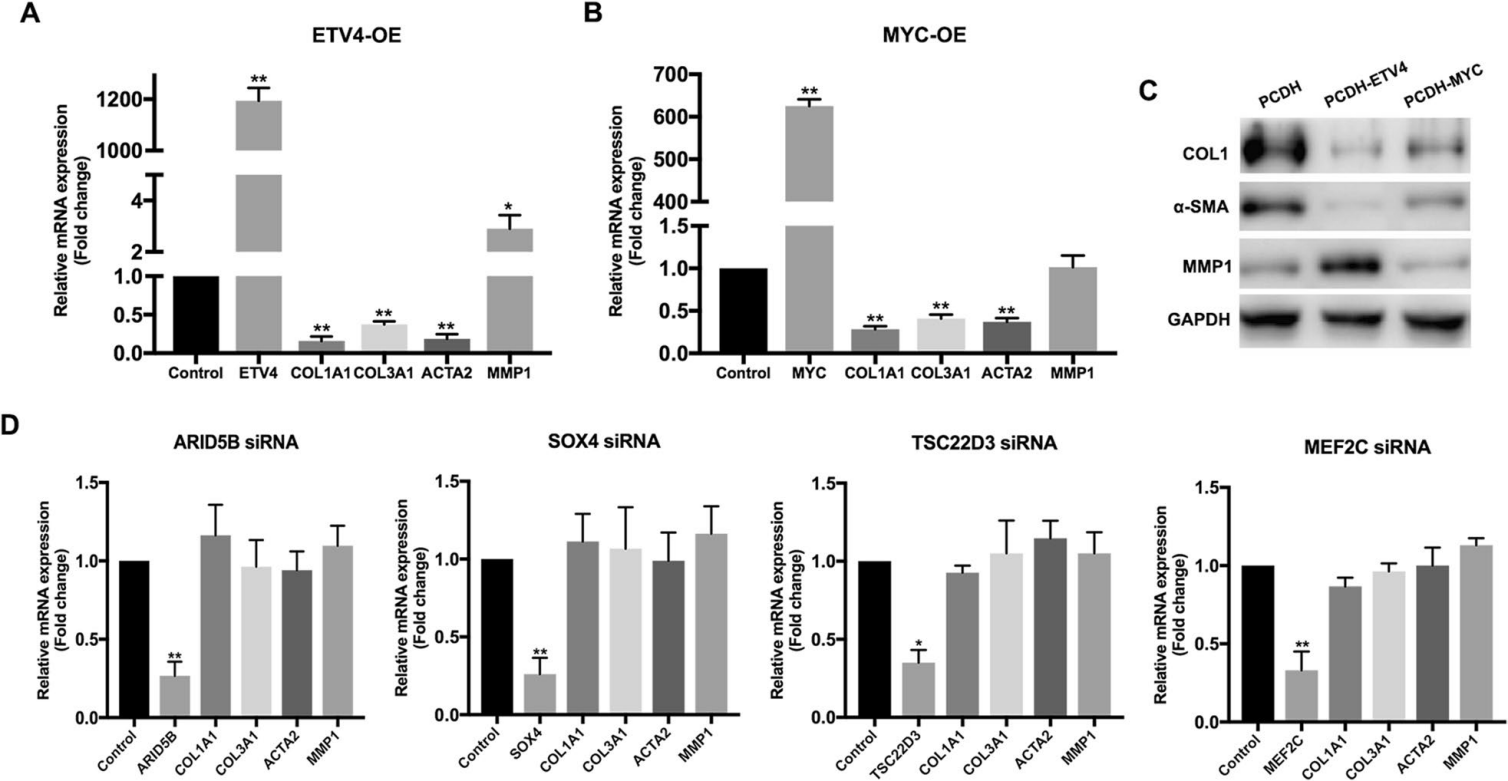
The overexpression and the knockdown of rescued TFs in HSFs. **A** The mRNA expression levels of representative fibrosis-related genes of HSFs after ETV4 overexpression were detected by qRT-PCR. **B** The mRNA expression levels of representative fibrosis-related genes of HSFs after MYC overexpression were detected by qRT-PCR. **C** The protein expression levels of representative fibrosis-related genes of HSFs after ETV4 and MYC overexpression were detected by western blot respectively. **D** The mRNA expression levels of representative fibrosis-related genes of HSFs after ARID5B, SOX4, TSC22D3, and MEF2C knockdown were detected by qRT-PCR respectively. Data are presented as the mean ± SD. *P < 0.05; **P < 0.01

To verify whether above rescued TFs were involved in the anti-fibrotic effect, we used siRNAs to knockdown *ARID5B*, *SOX4*, *TSC22D3*, *MEF2C*, and overexpress *ETV4* as well as *C-MYC* in HSFs, respectively. The knockdown and the overexpression of TFs in HSFs were confirmed by qRT-PCR (Fig. 4A, B, D). The gene expression levels of *COL1A1*, *COL3A1*, and *ACTA2* were significantly downregulated in *ETV4-* and *C-MYC*-overexpressed HSFs; however, *MMP1* upregulation was observed only in *ETV4-*overexpressed HSFs (Fig. 4A and B). Western blots showed the same expression changes of these fibrosis related genes, where *ETV4* overexpression remarkably suppressed the expression of COL1 and α-SMA and elevated MMP1 expression at protein level (Fig. 4C). Moreover, qRT-PCR results showed that knockdown of *ARID5B*, *SOX4*, *TSC22D3*, and *MEF2C* did not affect the expression of fibrogenesis-related genes in HSFs (Fig. 4D). Therefore, we proposed that ETV4 play the most significant role in the regulation of *COL1A1, COL3A1, ACTA2*, and *MMP1* in HSFs by microfat.

### Adipokines involved in the anti-fibrotic function of microfat were identified using proteomics

**Fig. 5:**
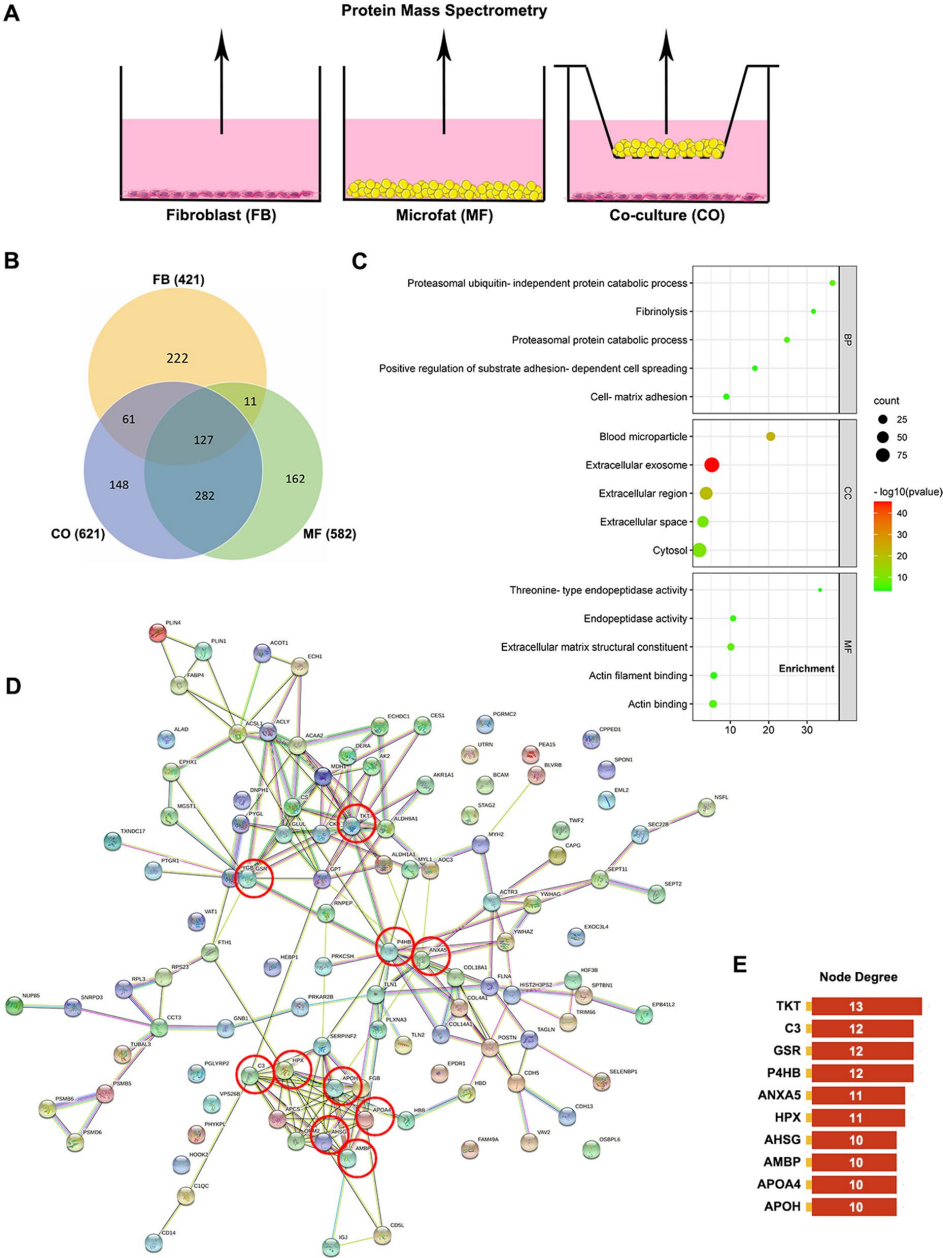
Mass spectrometric analysis of adipokines involved in the anti-fibrotic function of microfat. **A** Schematic diagram for the grouping design. **B** Venn diagram of the secretory proteins which was identified in the FB group, MF group, and CO group. **C** GO enrichment analysis of 282 microfat-secreted proteins. **D** The protein-protein interaction network of 282 microfat-secreted proteins. Red circles indicate the top 10 proteins ranking by node degree. **E** The top 10 proteins based on the node degree rank

Recent studies showed that proteins secreted by adipose tissue exhibit anti-inflammatory and anti-fibrotic effects. To screen the potential anti-fibrotic adipokines secreted by microfat, comparative secretome analyses were conducted in HSFs group (FB), microfat group (MF), and co-culture group (CO). Protein contents in the secretomes of the three groups were measured by the mass spectrometry (Fig. 5A). A total of 421, 582, and 621 proteins were identified in FB group, MF group, and CO group, respectively. Venn diagram classification showed that MF group and CO group shared 409 common proteins, of which 282 proteins were found only in MF group and CO group since the other 127 proteins were also present in the FB group (Fig. 5B). We hypothesized that anti-fibrotic proteins be secreted by co-cultured microfat, so the 282 proteins were further analyzed. Significantly enriched terms of the biological processes (BP), cell components (CC), and molecular functions (MF) of the 282 proteins were sorted according to *padj* value (Fig. 5C). The CC ontology showed that the 282 proteins were released into the extracellular region with microparticles and exosomes. Additionally, enriched MF and BP terms of the 282 proteins were mainly associated with extracellular matrix structural constituent, actin filament binding as well as cell-matrix adhesion and fibrinolysis, indicating that they might be closely relevant to the amelioration of hypertrophic scars.

Moreover, to investigate the interactions among the 282 proteins, we conducted the protein-protein interaction (PPI) networks using the String database, and the generated network showed proteins as nodes and interactions as colored lines linking them (Fig. 5D). The node degree was ranked according to the number of established links, and the top 10 degrees of proteins were shown (Fig. 5E). AHSG (Alpha 2-HS Glycoprotein), also known as fetuin-A, is a multifunctional protein secreted by both liver and adipose tissue; it came into our notice as it had been reported to attenuate renal fibrosis by antagonizing TGF-β signaling [23].

### Microfat-secreted fetuin-A decreases the expression of fibrosis-related genes via upregulating *ETV4* in HSFs

**Fig. 6:**
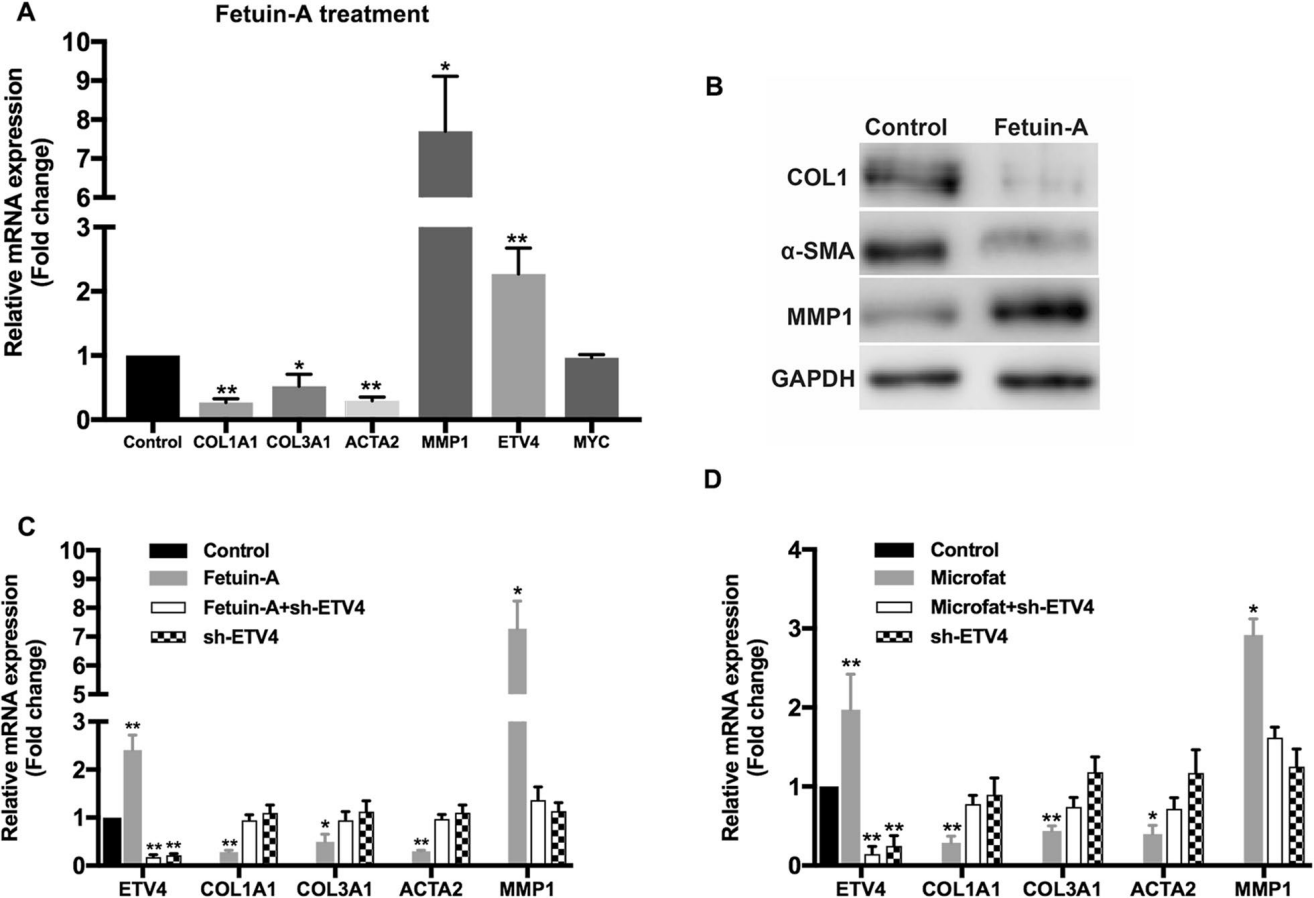
Fetuin-A decreases the expression of fibrosis-related genes of HSFs via upregulating ETV4. **A** The mRNA expression levels of representative fibrosis-related genes of HSFs after fetuin-A treatment were detected by qRT-PCR. **B** The protein expression levels of representative fibrosis-related genes of HSFs after fetuin-A treatment were detected by western blot. **C** The regulation of representative fibrosis-related genes in HSFs after fetuin-A treatment were hindered by sh-ETV4. **D** The regulation of representative fibrosis-related genes in HSFs co-cultured with microfat were hindered by sh-ETV4. Data are presented as the mean ± SD. *P < 0.05; **P < 0.01

To verify whether Fetuin-A was associated with the anti-fibrotic effect of microfat, HSFs were incubated with fetuin-A for 48 h and the expression of fibrosis-related genes were evaluated with qRT-PCR and western blot. Compared with the control group, fetuin-A treatment significantly reduced the gene expression of *COL1A1*, *COL3A1*, *ACTA2* but promoted *MMP1* expression at both mRNA and protein levels (Fig. 6A, B). Interestingly, the expression of *ETV4* was also significantly upregulated in HSFs incubated with fetuin-A, whereas no notable change was found in *C-MYC* expression.

Therefore, we hypothesized that fetuin-A might exert the anti-fibrotic effect on HSFs via regulating ETV4. We then knockdowned *ETV4*, which was followed by incubating HSFs with fetuin-A. The results showed that the changes of *COL1A1*, *COL3A1*, *ACTA2*, and *MMP1* induced by fetuin-A were hindered by sh-ETV4 (Fig. 6C), whereas sh-ETV4 alone showed no influence on the pathological expression of these fibrogenesis-related genes, suggesting that fetuin-A/ETV4 signaling play a specific role in the anti-fibrotic function of microfat. Moreover, when *ETV4* knockdown was followed by incubating HSFs with microfat, the decreased expression of *COL1A1*, *COL3A1*, and *ACTA2*, as well as the increased expression of *MMP1* in HSFs were also held back by sh-ETV4 (Fig. 6D). Together, our results demonstrated that fetuin-A/ETV4 signaling was the required pathway for the anti-fibrotic effect of microfat on HSFs.

## Discussion

Hypertrophic scarring caused by cutaneous injuries presents a global medical and economic burden, yet recently adipose tissue and fat-derived cells were successfully applied for clinical treatment. Sardesai et al. [24] firstly reported a prospective cohort study on dermal changes after fat grafting into cutaneous scars. The dermal elasticity as well as patients’ qualitative assessment of scar characteristics such as scar thickness and stiffness were both significantly improved over one year after the treatment of subdermal fat grafting. During the pathological process of hypertrophic scar formation, fibroblasts are activated to differentiate into myofibroblasts which express excessive fibrosis-related genes [1, 25]. In the current study, the anti-fibrotic effect of microfat on human hypertrophic scars was confirmed by both in vitro indirect co-culture experiments and in vivo mice models. We then identified the key TFs of co-cultured HSFs and the pivotal microfat-secreted proteins, which were involved in the anti-fibrotic effect of microfat. The results demonstrated that microfat-secreted fetuin-A could decrease the expression of fibrosis-related genes via upregulating *ETV4* expression in HSFs, which presents fetuin-A/ETV4 signaling as a potent anti-fibrotic pathway and provides attractive targets for future fibrotic disease treatment.

Our results showed that *ETV4* overexpression in HSFs resulted in a notable amelioration of fibrotic ECM genes of *COL1A1, COL3A1, ACTA2* and *MMP1*. ETV4 is an ETS transcription factor involved in a variety of biological processes, such as the regulation of cell migration, proliferation, differentiation, and apoptosis [26, 27]. Recent studies showed that TFs of the ETS family could cooperate with other transcription factors, such as AP1, SP1, and NF-κB, to promote the expression of matrix metalloproteases, which is closely related to the local invasion and distant metastasis of tumors [28–31]. This finding is also corroborated by our result that MMP1 was drastically upregulated in *ETV4-*overexpressed HSFs. Moreover, Akagi T et al. [32] reported that ETS-related TFs ETV4 and ETV5 are specifically expressed in undifferentiated embryonic stem (ES) cells; meanwhile, the expression of some stem-cell-related genes, including Tcf15, Gbx2, Zic3, and Lrh1, was dysregulated in Etv4/5 knock-out (dKO) ES cells, indicating that ETV4 and ETV5 are involved in maintaining the undifferentiated status of ES cells. ETV4 and ETV5 were also reported to play crucial roles in the stemness of primary salivary human stem/progenitor cells [33]. Our data showed that the expression level of transcription factor ETV4 was significantly lower in HSFs than in normal skin fibroblasts, while it was upregulated in HSFs co-cultured with microfat. Therefore, upregulated ETV4 might enhance the plasticity of HSFs during the reprogramming process of microfat on hypertrophic scars, which needs to be further clarified.

Moreover, we firstly demonstrate that fetuin-A secreted by microfat exerts an anti-fibrotic effect on HSFs as it downregulates the expression of fibrogenesis genes. Fetuin-A is a serum glycoprotein which is encoded by AHSG gene, which is mainly synthesized by the liver while can also be detected in adipose tissue, skeletal muscle, and skin [34–36]. The molecular structures of fetuin-A and TGF-βRII receptor are similar, with 18 to 19 shared amino acids in their sequences, and fetuin-A was thus considered as a natural antagonist of TGF-β signaling pathway [23]. Peterson et al. [37] reported that fetuin-A could inhibit the expression of TGF-β1 in liver stellate cells after 24 h of culture in vitro, and thus may be beneficial to alleviating hepatic fibrosis. Rudloff S et al. [38] identified fetuin-A as an evolutionary conserved HIF target gene, and further demonstrated that fetuin-A preserved kidney function through locally counteracting calcification, modulating macrophage polarization, and attenuating inflammation and fibrosis. Fetuin-A attenuated the hypoxia-induced expression of fibrosis marker genes including *ACTA2* and *COL3A1* by antagonizing TGF-β signaling; besides, fetuin-A supplementation could reduce the expression of *COL1A1* and *COL3A1* in ischemia-reperfusion injury kidneys. Fetuin-A has been proved to have effects on inhibiting inflammatory response and fibrosis, promoting wound healing, and so on [39]. Wang et al. [40] showed that fetuin-A abounded in ovine fetal skin, and might be intrinsically responsible for the scarless healing observed in the fetus; besides, fetuin-A could markedly enhance wound closure in primary keratinocytes by increasing cell migration. Fetuin-A possessed great potential to improve the outcome of burn wound, not only by promoting wound closure, but may also by getting involved in the inflammatory response and myofibroblast formation [41].

In this present study, ETV4 was dramatically upregulated in HSFs incubated with fetuin-A, and sh-ETV4 could hinder the anti-fibrotic effect of fetuin-A on HSFs, indicating that microfat alleviates hypertrophic scars via fetuin-A/ETV4 axis. This study provides an important theoretical basis for the improvement of hypertrophic scars by fat grafting, and a direction for the development of new therapeutic methods in clinical practice as well. Our shortcoming lies in that the molecular mechanism still needs to be further studied, and there is still a lack of in vivo experimental verification and long-term observation. To sum up, we confirmed the anti-scar effect of microfat in both in vivo scar models and in vitro co-culture experiments. Furthermore, we demonstrated that microfat-secreted fetuin-A exerts a pivotal effect on the anti-fibrotic regulation of HSFs through upregulating the expression of the transcription factor ETV4.

## Conclusions

Microfat grafting exerts a remarkable anti-fibrotic on human hypertrophic scars both in vitro experiments and in vivo mice scar models. The upregulation of transcription factor ETV4 is essential for decreasing the expression of fibrosis-related genes. Fetuin-A secreted by microfat also suppress the fibrotic character of HSFs. Lastly, the change of fibrosis-related genes expression owing to fetuin-A treatment were hindered by downregulating ETV4. These findings indicate that fetuin-A/ETV4 signaling was the required pathway for the anti-fibrotic effect of microfat on human hypertrophic scars.

## Supplementary Information

**Additional file 1. The ORF sequences of ETV4 and C-MYC, siRNAs sequences, and shRNA sequence for ETV4**

**Additional file 2. The primer sequences for qRT-PCR**

## Data Availability

The datasets used and/or analyzed during this study are available from the corresponding author on reasonable request.

## Abbreviations

AHSG: Alpha 2-HS glycoprotein
α-SMA: Alpha-smooth muscle actin
DEG: Differential expressed gene
FPKM: Fragments per kilobases per million reads
GSEA: Gene set enrichment analysis
HSF: Hypertrophic scar fibroblast
MMP-1: Matrix metalloprotease 1
ORF: Open reading frame
PBS: Phosphate-buffered saline solution
PPI: Protein–protein interaction
shRNA: Short hairpin RNA
siRNA: Small interfering RNA
SVF: Stromal vascular fraction
TF: Transcription factor
Tgf-β1: Transforming growth factor beta 1
Vegf: Vascular endothelial growth factor
