# Supplementary material for: Microfat exerts an anti-fibrotic effect on human hypertrophic scar via fetuin-A/ETV4 axis
Source: J Transl Med. 2023 Mar 31;21:231. doi: 10.1186/s12967-023-04065-y (PMC10064544; doi:10.1186/s12967-023-04065-y)
Supplement: Supplementary file 1 — Additional file 1. The ORF sequences of ETV4 and C-MYC, siRNAs sequences, and shRNA sequence for ETV4 [file 12967_2023_4065_MOESM1_ESM.docx]

**ETV4 ORF**

atg gagcggagga tgaaagccgg atacttggac

241 cagcaagtgc cctacacctt cagcagcaaa tcgcccggaa atgggagctt gcgcgaagcg

301 ctgatcggcc cgctggggaa gctcatggac ccgggctccc tgccgcccct cgactctgaa

361 gatctcttcc aggatctaag tcacttccag gagacgtggc tcgctgaagc tcaggtacca

421 gacagtgatg agcagtttgt tcctgatttc cattcagaaa acctagcttt ccacagcccc

481 accaccagga tcaagaagga gccccagagt ccccgcacag acccggccct gtcctgcagc

541 aggaagccgc cactccccta ccaccatggc gagcagtgcc tttactccag tgcctatgac

601 ccccccagac aaatcgccat caagtcccct gcccctggtg cccttggaca gtcgccccta

661 cagccctttc cccgggcaga gcaacggaat ttcctgagat cctctggcac ctcccagccc

721 caccctggcc atgggtacct cggggaacat agctccgtct tccagcagcc cctggacatt

781 tgccactcct tcacatctca gggagggggc cgggaacccc tcccagcccc ctaccaacac

841 cagctgtcgg agccctgccc accctatccc cagcagagct ttaagcaaga ataccatgat

901 cccctgtatg aacaggcggg ccagccagcc gtggaccagg gtggggtcaa tgggcacagg

961 tacccagggg cgggggtggt gatcaaacag gaacagacgg acttcgccta cgactcagat

1021 gtcaccgggt gcgcatcaat gtacctccac acagagggct tctctgggcc ctctccaggt

1081 gacggggcca tgggctatgg ctatgagaaa cctctgcgac cattcccaga tgatgtctgc

1141 gttgtccctg agaaatttga aggagacatc aagcaggaag gggtcggtgc atttcgagag

1201 gggccgccct accagcgccg gggtgccctg cagctgtggc aatttctggt ggccttgctg

1261 gatgacccaa caaatgccca tttcattgcc tggacgggcc ggggaatgga gttcaagctc

1321 attgagcctg aggaggtcgc caggctctgg ggcatccaga agaaccggcc agccatgaat

1381 tacgacaagc tgagccgctc gctccgatac tattatgaga aaggcatcat gcagaaggtg

1441 gctggtgagc gttacgtgta caagtttgtg tgtgagcccg aggccctctt ctctttggcc

1501 ttcccggaca atcagcgtcc agctctcaag gctgagtttg accggcctgt cagtgaggag

1561 gacacagtcc ctttgtccca cttggatgag agccccgcct acctcccaga gctggctggc

1621 cccgcccagc catttggccc caagggtggc tactcttact ag

**MYC ORF**

ctggattttt ttcgggtagt ggaaaaccag cctcccgcga

1201 cgatgcccct caacgttagc ttcaccaaca ggaactatga cctcgactac gactcggtgc

1261 agccgtattt ctactgcgac gaggaggaga acttctacca gcagcagcag cagagcgagc

1321 tgcagccccc ggcgcccagc gaggatatct ggaagaaatt cgagctgctg cccaccccgc

1381 ccctgtcccc tagccgccgc tccgggctct gctcgccctc ctacgttgcg gtcacaccct

1441 tctcccttcg gggagacaac gacggcggtg gcgggagctt ctccacggcc gaccagctgg

1501 agatggtgac cgagctgctg ggaggagaca tggtgaacca gagtttcatc tgcgacccgg

1561 acgacgagac cttcatcaaa aacatcatca tccaggactg tatgtggagc ggcttctcgg

1621 ccgccgccaa gctcgtctca gagaagctgg cctcctacca ggctgcgcgc aaagacagcg

1681 gcagcccgaa ccccgcccgc ggccacagcg tctgctccac ctccagcttg tacctgcagg

1741 atctgagcgc cgccgcctca gagtgcatcg acccctcggt ggtcttcccc taccctctca

1801 acgacagcag ctcgcccaag tcctgcgcct cgcaagactc cagcgccttc tctccgtcct

1861 cggattctct gctctcctcg acggagtcct ccccgcaggg cagccccgag cccctggtgc

1921 tccatgagga gacaccgccc accaccagca gcgactctga ggaggaacaa gaagatgagg

1981 aagaaatcga tgttgtttct gtggaaaaga ggcaggctcc tggcaaaagg tcagagtctg

2041 gatcaccttc tgctggaggc cacagcaaac ctcctcacag cccactggtc ctcaagaggt

2101 gccacgtctc cacacatcag cacaactacg cagcgcctcc ctccactcgg aaggactatc

2161 ctgctgccaa gagggtcaag ttggacagtg tcagagtcct gagacagatc agcaacaacc

2221 gaaaatgcac cagccccagg tcctcggaca ccgaggagaa tgtcaagagg cgaacacaca

2281 acgtcttgga gcgccagagg aggaacgagc taaaacggag cttttttgcc ctgcgtgacc

2341 agatcccgga gttggaaaac aatgaaaagg cccccaaggt agttatcctt aaaaaagcca

2401 cagcatacat cctgtccgtc caagcagagg agcaaaagct catttctgaa gaggacttgt

2461 tgcggaaacg acgagaacag ttgaaacaca aacttgaaca gctacggaac tcttgtgcgt

2521 aa

| ARID5B siRNA | F: CAUUCAGUGGUGUUAAGGATT |
| --- | --- |
|  | R: UCCUUAACACCACUGAAUGTT |
| SOX4 siRNA | F: GCGACAAGAUCCCUUUCAUTT |
|  | R: AUGAAAGGGAUCUUGUCGCTT |
| TSC22D3 siRNA | F: GGAGAAGAACUCCCAGCUATT |
|  | R: UAGCUGGGAGUUCUUCUCCTT |
| MEF2C siRNA | F: GGUAACUUGAACAAGAAUATT |
|  | R: UAUUCUUGUUCAAGUUACCTT |
| CTRL siRNA | F: UUCUCCGACAGUGUCACGUTT |
|  | R: ACGUGACACUGUCGGAGAATT |

| ETV4-shRNA | CCGG-AGCGTTACGTGTACAAGTTTG-CTCGAGCAAACTTGTACACGTAACGCT-TTTTTG |
| --- | --- |
