# Supplementary material for: Microfat exerts an anti-fibrotic effect on human hypertrophic scar via fetuin-A/ETV4 axis
Source: J Transl Med. 2023 Mar 31;21:231. doi: 10.1186/s12967-023-04065-y (PMC10064544; doi:10.1186/s12967-023-04065-y)
Supplement: Supplementary file 2 — Additional file 2. The primer sequences for qRT-PCR [file 12967_2023_4065_MOESM2_ESM.docx]

| COL1A1 | F: GGGATTCCCTGGACCTAAAG |
| --- | --- |
|  | R: GGAACACCTCGCTCTCCA |
| COL3A1 | F: CTGGACCCCAGGGTCTTC |
|  | R: CATCTGATCCAGGGTTTCCA |
| ACTA2 | F: AAAGCAAGTCCTCCAGCGTT |
|  | R: GCCCTTCCACAATGCCAAAG |
| MMP1 | F: CTGAAAGTGACTGGGAAACC |
|  | R: GACAAACTGAGCCACATCAG |
| ETV4 | F: GTCTGCGTTGTCCCTGAGAA |
|  | R: AGCTTGAACTCCATTCCCCG |
| MYC | F: TAGTGGAAAACCAGCAGCCT |
|  | R: CTCCTCCTCGTCGCAGTAGA |
| ARID5B | F: AGCAACATGGCCCAGTGATA |
|  | R: TGGCTCAGCATTCTTCTGGT |
| SOX4 | F: TGTGTTCAGGTTTATAGCTGTTGTG |
|  | R: AGACGTCCTAGTGCCTGTCA |
| TSC22D3 | F: CTTGCTGCTGCAACCCTTTC |
|  | R: AGTGAATGGTGGGTTTGGCA |
| MEF2C | F: AGACCCTTTAATAAGTGAGTGCCA |
|  | R: TCTTCACATTCCAAGAGAAGGAA |
| MYBL2 | F: GATGATGTCCACACTGCCCA |
|  | R: ATACCTGACAGGGTGAGGCT |
| RFX8 | F: AAGAAAGCCCTGGCAAGTGA |
|  | R: TGTGAAGACAGACAGCTGAGG |
| POU2F2 | F: GGACCTTACCGTTGTCCCAA |
|  | R: CCCCACAGCTGAGGATAAGG |
| TCF19 | F: CCAGAGCCTGTCTTTCAGCA |
|  | R: GTAATGGTCCTCTGTCCCGC |
| MAFB | F: GTCCTGCATCAGAAACGAGC |
|  | R: CAATTTCTGTTGCGGCAGGT |
| IRX5 | F: TGTCCGACATTTAACGCGGG |
|  | R: ACTGCCAAGGCCATGTTTTT |
| ID2 | F: GACACAAGCCTACTGAATGCTG |
|  | R: AGAGTTCACAAGGTTTCACTCA |
| GAPDH | F: TTGCCCTCAACGACCACTTT |
|  | R: TGGTCCAGGGGTCTTACTCC |
